# Microbial keratitis-induced endophthalmitis: incidence, symptoms, therapy, visual prognosis and outcomes

**DOI:** 10.1186/s12886-018-0777-3

**Published:** 2018-05-03

**Authors:** Daniel Zapp, Daria Loos, Nikolaus Feucht, Ramin Khoramnia, Tamer Tandogan, Lukas Reznicek, Christian Mayer

**Affiliations:** 1 Department of Ophthalmology, Klinikum rechts der Isar, Technical University of Munich, Ismaninger Str. 22, 81675 Munich, Germany; 2 Department of Ophthalmology, University of Heidelberg, Heidelberg, Germany

**Keywords:** Endophthalmitis, Keratitis, Enucleation, Infection, Corneal ulcer

## Abstract

**Background:**

To evaluate symptoms, therapies and outcomes in rare microbial keratitis-induced endophthalmitis.

**Methods:**

Retrospective study with 11 patients treated between 2009 and 2014. Clinical findings, corneal diseases, history of steroids and trauma, use of contact lenses, number and type of surgical interventions, determination of causative organisms and visual acuity (VA) were evaluated.

**Results:**

The incidence of transformation from microbial keratitis to an endophthalmitis was 0.29% (*n* = 11/3773). In 90.9% (*n* = 10/11), there were pre-existent eyelid and corneal problems, in 45.5% (*n* = 5/11) rubeosis iridis with increased intraocular pressure and corneal decompensation, and in 18.2% (*n* = 2/11), ocular trauma. Specimens could be obtained in 10 of 11 samples: 33.3% of those 10 specimens were Gram-positive coagulase-negative Staphylococci (*n* = 3/10) or Gram-negative rods (n = 3/10) and 10.0% *Staphylococcus aureus* (*n* = 1/10). In 30% (n = 3/10), no pathogens were identifiable. 72.7% (*n* = 8/11) of all keratitis-induced endophthalmitis were treated with vitrectomy and 9.1% (n = 1/11) with amniotic-membrane transplantation. In 27.3% (n = 3/11) the infected eye had to be enucleated – 18.2% (*n* = 2/11) primarily, 9.1% (n = 1/11) secondarily. No patient suffered from sympathetic ophthalmia. The median initial VA was 2.1 logMAR (*n* = 11/11). At one month, median VA was 2.0 logMAR (*n* = 7/11), after three months 2.0 logMAR (*n* = 6/11), and after one year 2.05 logMAR (n = 6/11). The change in VA was not significant (*p* > 0.99). 36.4% (*n* = 4/11) of the cases resulted in blindness.

**Conclusions:**

The overall outcome is poor. Enucleation should be weighed against the risk of local and systemic spread of the infection, prolonged rehabilitation and sympathetic ophthalmia.

## Background

Infectious endophthalmitis is a rare and severe inflammation of the intraocular tissues and fluids of the eye, involving the anterior and posterior eye segment and the adjacent sclera [1, 2]. Endophthalmitis of either form, exogenous or endogenous, can lead to a significant reduction of visual acuity and, at worst, result in a loss of the affected eye [3]. Exogenous endophthalmitis is caused by microbial pathogens that enter the eye after surgery or trauma, or infiltrate through the surface. Microbial keratitis-induced endophthalmitis is a sight-threatening disease, often bearing the worst possible visual outcome [4].

Microbial keratitis-induced endophthalmitis is uncommon (0.5% [5] to 6.1% [3, 6–9]), especially in an otherwise healthy eye [5, 8]. Different treatment options in severe keratitis exist, such as fortified local and/or systemic antibiotics, crosslinking and keratoplasty à chaud in contrast to intravitreal or vitrectomy in microbial keratitis-induced endophthalmitis. Literature shows a wide spread in prevalence rates, most probably due to the lack of a common definition of microbial keratitis cases, let alone microbial keratitis-induced endophthalmitis. Inconsistencies in routinely taking swabs in cases of initially mild keratitis that later develop into endophthalmitis might further cloud a correct estimation of microbial association and detection rates.

**Fig. 1:**
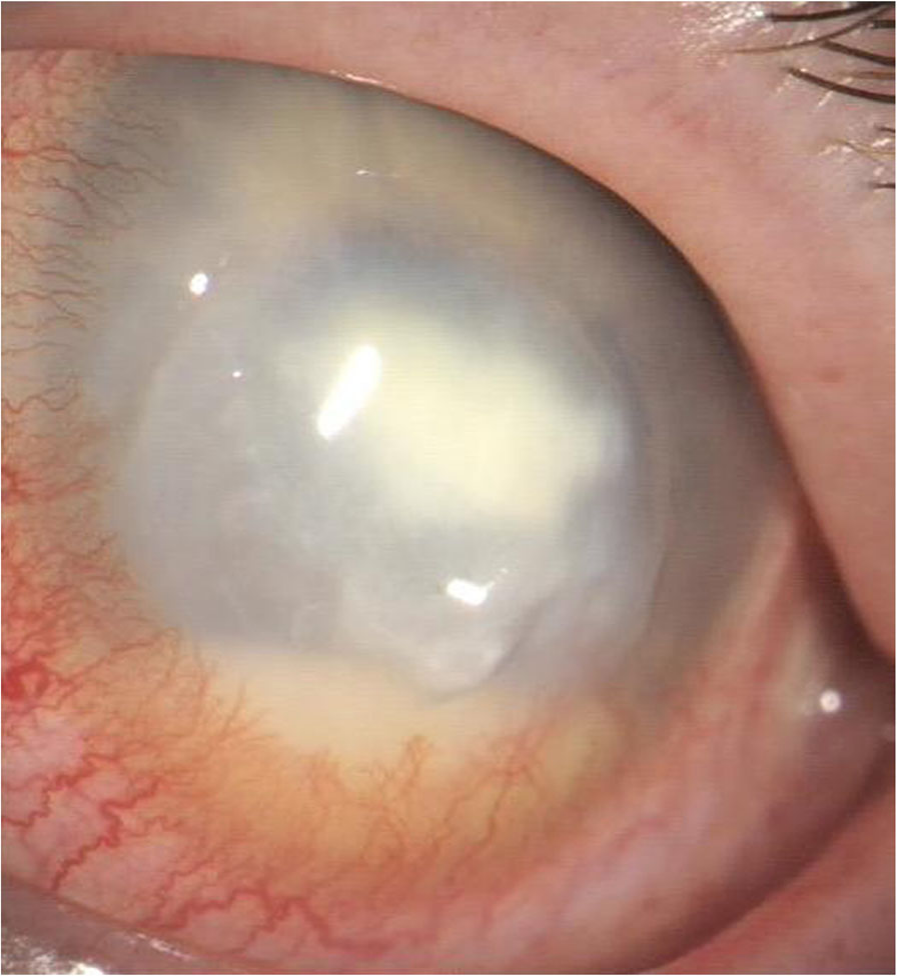
Clinical image of microbial keratitis-induced endophthalmitis. The diagnosis is determined by clinical findings: visual acuity decrease, pain, hypopyon and vitreous body infiltration

In the majority of cases, endophthalmitis is diagnosed clinically, [7, 10] with typical symptoms of loss of vision, photophobia and pain (Fig. 1).

Clinical signs consist of conjunctival injection as well as infiltrates, oedema, opacities and endothelium precipitates on the cornea. Moreover, it might be possible to detect anterior chamber flare, cells, fibrin or hypopyon, altered pupil, vitreous infiltrates, periphlebitis, retinal haemorrhages, Roth’s spots or reduced or even loss of fundus visualization [2, 11, 12]. Ultrasound can be of substantial aid in cases with reduced posterior visualization; however, it might also be misleading at times and its limited local availability should not lead to a delay in starting the treatment if sufficient clinical suspicion is raised. This is supported by the fact that the most common form of uncomplicated microbial keratitis holds a rather good prognosis when treated correctly and in a timely manner, contrary to endophthalmitis with a severely reduced visual prognosis of light-perception or worse [13, 14].

Pre-existing dry eye disease, blepharo-conjunctivitis, corneal perforation, recent trauma or surgery, immunosuppression and local or systemic steroid therapy have all been identified as risk factors of progression to endophthalmitis in initially corneal infections [3, 5].

Common forms of treatment include anti-inflammatory and antibiotic drugs, corneal scraping and vitrectomy [15, 16]. During surgery, samples may be taken for a microbiological or virological analysis followed by intravitreal antibiotic treatment.

The goal of this retrospective analysis was to evaluate symptoms, therapies and outcomes in rare but sight- and eye-threatening microbial keratitis-induced endophthalmitis in our department of ophthalmology.

## Methods

In a retrospective analysis, all patients treated with endophthalmitis between December 2006 and December 2011 in the Department of Ophthalmology, Klinikum rechts der Isar, Technical University of Munich, Germany, were identified using the computer software program clinical information system I.S.H. med^®^ (SAP^®^ SE, Walldorf, Germany). From this collective, all cases of microbial keratitis-induced endophthalmitis were extracted, pseudonymized and included in this study. None of these patients had relevant ophthalmic preconditions to be excluded from the study. The following patient data was identified: age, gender, localisation, clinical findings and subjective symptoms, surgeries, trauma, prediagnosed corneal and eyelid diseases, history of steroids, previous history of trauma, and use of contact lenses. The study was conducted in accordance with the tenets of the Declaration of Helsinki and approved by the internal Institutional Review Board of Klinikum rechts der Isar, Technical University of Munich.

For all patients, the time interval between the onset of symptoms and diagnosis was analysed. In all cases, ultrasound examination was performed to confirm the clinical diagnosis of endophthalmitis. Corneal swabs were taken in all cases with diagnosed endophthalmitis upon intial presentation and directly processed by the on-site Microbiological Department by specimen cultivation. Broad-spectrum antibiotics were initiated according to the Magdeburg treatment regimen: [17] vancomycin (1 g bid intravenously) and ceftazidime (2 g tid intravenously) were administered to cover Gram-positive pathogens and Gram-negative pathogens respectively. Systemic steroids (prednisolone, 1-2 mg/kg) were added to the therapy one day after starting the antibiotic therapy to limit further tissue destruction by antigens and cytokines released from infiltrating leukocytes [2, 17]. Intensive topical moxifloxacin and a fixed combination of polymyxin B, neomycin and gramicidin eye drops were administered initially ¼ to ½ hourly and tapered to hourly by day 2. Antibiotic therapy was adjusted after receiving the appropriate antibiogram [6].

Anterior chamber and vitreous sample aspiration were performed in eyes with increasing hypopyon and affected vitreous shown in ultrasound examination. Following removal of the vitreous including gathering of microbial samples, an intravitreal and intracameral therapy with vancomycin (0.05 ml: 20 mg/ml) and ceftazidime (0.1 ml; 2.25 mg/ml) were applied. Postoperatively, subconjunctival and topical broad-spectrum antibiotics (gentamycin 40 mg/1 ml) and steroids (dexamethasone 4 mg/ml) were administered [17].

Amniotic membrane transplants were performed in cases of severe surface defects. Primary enucleation was only performed in cases of painful amaurosis with no light perception.

Best-corrected visual acuity (BCVA) was assessed on initial presentation (day 0), after one month (1 m), three months (3 m) and after one year (1y). Complications defined as retinal detachment, recurrence of infection, lack of improvement, enucleation and blindness were evaluated for a one-year follow-up time period.

A statistical comparison depending on the aetiology of keratitis-induced endophthalmitis (Kruskal-Wallis test), the clinical findings (Mann-Whitney U test) and the form of therapy (Kruskal-Wallis test) was performed. A *p*-value of < 0.05 was considered statistically significant.

## Results

**Fig. 2:**
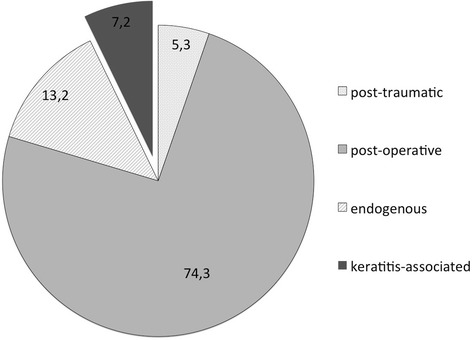
Prevalence of microbial keratitis-induced endophthalmitis in comparison to all triggers of endophthalmitis (in %)

Altogether, 152 eyes of 149 individuals presented with endophthalmitis to the Department of Ophthalmology, Technical University of Munich, Germany between December 2006 and December 2011. In 74.3% (*n* = 113/152) of our cases, endophthalmitis originated from previous surgery, 5.3% (*n* = 8/152) had a history of recent trauma and in 13.2% (*n* = 20/152) an endogenous endophthalmitis was diagnosed. In 7.2% (*n* = 11/152), the endophthalmitis developed from an initial microbial keratitis (Fig. 2).

In that same time period, overall 3773 patients were recorded with diagnosed microbial keratitis who were treated at least with topical antibiotics. The transformation rate from microbial keratitis to an endophthalmitis in our collective was 0.29% (*n* = 11/3773).

In 90.9% (*n* = 10/11) of patients with keratitis-induced endophthalmitis, a pre-existence of eyelid and corneal problems was observed. Overall, 63.6% (*n* = 7/11) had a history of topical or systemic steroid therapy. Rubeosis iridis was present in 45.5% (*n* = 5/11), along with increased intraocular pressure (IOP) and corneal decompensation. In all of these cases, the IOP decompensation was caused by either retinal vein occlusion or diabetic retinopathy. In 18.2% (*n* = 2/11) of the microbial-induced endophthalmitis cases, patients had a history of a recent corneal trauma. None of the patients were contact-lens wearers.

The mean age of all patients with keratitis-induced endophthalmitis was 67 years (range 32-89), while the mean age of all patients with endophthalmitis was 70 years (range 17-89). About half of all patients with keratitis-induced endophthalmitis (45.5%, *n* = 5/11) were female. The right eye was affected more frequently (63.6%, *n* = 7/11) than the left. The timespan from first onset of patient reported complaints to diagnosis was four days (range 1-360 days). The patient with almost one year time to diagnosis had fluctuations in the severity of his symptoms and inflammation and was externally treated with antibiotics of varying extent and route before final exacerbation led to admission. All patients (*n* = 11/11) presented with a clinically “red eye”, a reduction in visual acuity was initially present in 36.4% (*n* = 4/11). Pain was reported in 72.7% (*n* = 8/11), and a hypopyon could be detected in 90.9% (10/11). The fundus red reflex was lost in all cases (*n* = 11/11).

**Fig. 3:**
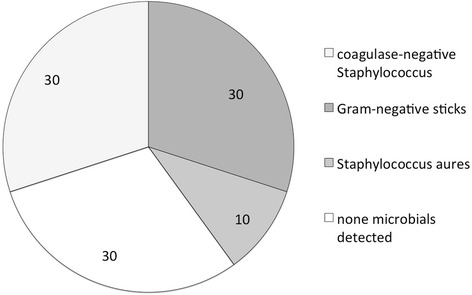
Microbial detection in microbial keratitis-induced endophthalmitis (percentage and number). Seven of 11 specimens revealed microbials

Microbiological samples could be obtained in 90.0% (*n* = 10/11) for causative organisms. Of those, 33.3% were Gram-positive coagulase-negative Staphylococci (*n* = 3/10) or Gram-negative rods (n = 3/10) respectively and 10.0% *Staphylococcus aureus* (n = 1/10). In 30% (*n* = 3/10), no pathogens were identifiable (Fig. 3).

Vitrectomy was performed in 72.7% (*n* = 8/11), primary enucleation in 18.2% (*n* = 2/11) and amniotic membrane transplantation in 9.1% (*n* = 1/11) of all keratitis-induced endophthalmitis cases.

**Fig. 4:**
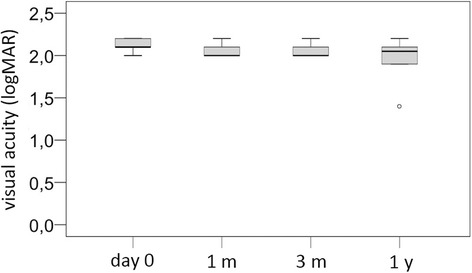
Development of visual acuity (logMAR) in microbial keratitis-induced endophthalmitis: Day 0 = initial presentation, 1 m = 1 month, 3 m = 3 months, 1y = 1 year: at no time was there a statistically significant improvement in visual acuity (all *p* > 0.05 respectively)

The median initial BCVA was 2.1 logMAR (*n* = 11/11; range 2.0-2.2). At one month, the median BCVA was 2.0 logMAR (*n* = 7/11; range 2.0-2.2), after three months 2.0 logMAR (*n* = 6/11; range 2.0-2.2) and after one year 2.05 logMAR (n = 6/11; range 1.4-2.2). The change in BCVA from baseline was not significant over time (*p* > 0.99) (Fig. 4).

Overall, 90.9% (*n* = 10/11) of all patients in the keratitis-induced endophthalmitis group became legally blind in the affected eye and 36.4% (*n* = 4/11) resulted in amaurosis with no light perception.

**Fig. 5:**
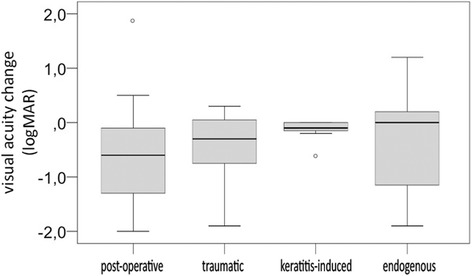
Visual acuity change in logMAR in the four different endophthalmitis aetiologies: post-operative, traumatic, microbial keratitis-induced and endogenous

The mean change in BCVA in the keratitis-induced endophthalmitis collective was − 0.1 logMAR, whereas the BCVA in the other endophthalmitis aetiologies were: − 0.6 logMAR in the post-operative, − 0.3 logMAR in the traumatic and ± 0 logMAR in the endogenous subgroup (*p* = 0.052; Fig. 5).

During the one-year follow-up, we did not observe any occurrences of retinal detachment. One patient (9.1%, n = 1/11) experienced a recurrence of endophthalmitis. Overall, 27.3% (*n* = 3/11) ended up in an enucleation (two of them were primary enucleations). No patient suffered from sympathetic ophthalmia. One patient died during the follow-up period from chronic cardiovascular disease, whereby a connection to keratitis-induced endophthalmitis remained unlikely.

## Discussion

In our examined study population, patients suffering from microbial keratitis-induced endophthalmitis originating from initial lesions of the corneal surface constituted the third largest group of all endophthalmitis aetiologies. Accounting for 7.2% of all cases of endophthalmitis at our clinic, this group was substantial in comparison to published data [3, 7]. This may be partially due to the general focus of our clinic on the anterior segment as well as the fact that in the urban area of our university clinic setting, a high density of posterior segment surgeons are commonly available. Therefore, in our study the postoperative endophthalmitis group might have been underrepresented.

In a healthy eye, eyelid, tear-film, epithelium, stroma and an intact descemet membrane offer protection against intraocular infections. In our study population of keratitis-induced endophthalmitis, 18.2% of patients had a positive history of recent corneal surface trauma; chronic inflammatory eyelid or corneal alterations were pre-existent in 90.9% of the group. It would have been interesting to compare those patients to matching control groups with same pre-existing risk factors but no initial keratitis. However, an external infection progressing to endophthalmitis by bypassing the cornea would be is an even rarer disease aside from special anatomical exceptions like e.g. glaucoma tubes or severe scleritis.

We found a progress rate from microbial keratitis to an endophthalmitis in 0.29%. A review of currently available literature illustrates that, in general, 0.5% [5] up to 6.1% [9] of corneal ulcers seem to progress and end up in endophthalmitis.

In about 80% of these cases, local or systemic steroids had previously been used, posing a well-known risk factor for keratitis-induced endophthalmitis [5]. On the other hand, the Steroids for Corneal Ulcers Trial (SCUT), a double-masked placebo controlled randomized study, raised no safety concerns in its 500 cases of keratitis treated with or without topical steroids. None of the patients progressed to endophthalmitis proving the potential benefit of steroids under appropriate use and antibiotic coverage [18].

Immune dysfunction, fungal keratitis, keratitis next to ​​a recent surgical wound and corneal penetration are also considered as potentially predisposing factors [5, 8]. Since none of these entities occurred in our study group, these risk factors could not be verified in our study. However, 45% of the patients had rubeosis iridis with secondary angle closure glaucoma causing corneal decompensation. Another study confirmed our findings and also reported a strong association of glaucoma and corneal oedema with secondary corneal ulcers in their patients [19]. Pathophysiologically, one might argue that elevated intraocular pressure levels and induced damage to the corneal endothelial cells [20] lead to decompensation and secondary oedema [21]. By decompensation and swelling of the cornea, tight junctions and microbiological barriers break down, allowing the keratitis to spread more easily and progress to a keratitis-induced endophthalmitis faster than in an uncompromised cornea.

In keratitis-induced endophthalmitis, the latency to definitive diagnosis can be expected to be higher than in other endophthalmitis entities [11]. This might be due to pre-existing minor visual loss and unremarkable pain caused by often associated chronic eyelid and corneal diseases. Therefore, patients are mostly accustomed to both to some extent, leading to a deferred consultation of an ophthalmologist.

On the other hand, keratitis-induced endophthalmitis could be rather difficult to diagnose: the frequent loss of the red-reflex may be related to intraocular inflammation itself, but could also result from the underlying corneal diseases and the frequent incidence of a hypopyon since both factors reduce general insight into the eye.

The spectrum of causative organisms included Gram-positive coagulase-negative Staphylococci (30%) and Gram-negative rods (30%). Other studies also found fungi as frequent pathogens in keratitis-induced endophthalmitis [5, 8].

However, it remains unclear up to date whether the final outcome is more determined by the patient’s comorbidities or the causative organisms. Fungi appear to show higher rates of progression to endophthalmitis and their prognosis is among the worst. Difficulties in cultivation, treatment availability and their ability to penetrate otherwise intact corneas potentially contribute to this [22]. In our study, we were unable to support these findings since we had no cases of proven fungal keratitis.

Patients with keratitis-induced endophthalmitis initially achieved a median visual acuity of 2.1 logMAR, which hardly improved to 2.05 logMAR within the first year. They therefore presented with a very poor visual outcome and prognosis. BCVA showed no significant changes at any of the follow-up intervals (all *p* > 0.05, median difference − 0.1 logMAR). In 27.3% of our keratitis-induced endophthalmitis cases, the infected eye had to be primarily or secondarily enucleated. Other studies also show the typically reduced prognosis in keratitis-induced endophthalmitis, [5, 8] with high rates of enucleation or evisceration [19, 23, 24]. Predisposing chronic corneal pathology or advanced secondary glaucoma with further reduced prognosis and treatment success rates may be at least partially responsible for this association. In respect to this extremely low visual prognosis, a primary enucleation or evisceration especially in devastating cases with pre-existing ocular pathologies must be considered a valid option since it has been shown that the risk of sympathetic ophthalmia is increased in keratitis-induced endophthamitis [25].

Therefore, keratitis-induced endophthalmitis represents one of the severest ophthalmic entities. It often results in poor visual outcomes despite extensive treatment. While most cases of keratitis-induced endophthalmitis entail a positive history of predisposing ophthalmic risk factors, the range of established risk factors or comorbidities differs from the cases with keratitis alone [24]. Since severe isolated keratitis mostly presents with similar clinical features, at least initially, distinguishing it from the visually far more endangering posterior endophthalmitic form can be crucial if only to preserve the eye as such.

## Conclusions

Microbial induced keratitis is a rare disease with variable presentation and course. Only 0,29% of initial keratitis cases progressed to endophthalmitis. The overall outcome of microbial keratitis-induced endophthalmitis is very poor, including high rates of enucleation and evisceration. The decision for enucleation or evisceration should be considered carefully in order not to endanger patients’ health by risk of systemic and local spread infection, prolonged rehabilitation and danger of sympathetic ophthalmia.

## Abbreviations

BCVA: Best-corrected visual acuity
bid: Twice a day
IOP: Intraocular pressure
logMAR: Logarithm of the Minimum Angle of Resolution
m: Month
tid: Three times daily
y: Year
